# Environmental Health-Related Policies and Practices of Oklahoma Licensed Early Care and Education Programs: Implications for Childhood Asthma

**DOI:** 10.3390/ijerph18168491

**Published:** 2021-08-11

**Authors:** Cassandra D. Querdibitty, Bethany Williams, Marianna S. Wetherill, Susan B. Sisson, Janis Campbell, Mary Gowin, Lancer Stephens, Alicia L. Salvatore

**Affiliations:** 1 Department of Health Promotion Sciences, Hudson College of Public Health, University of Oklahoma Health Sciences Center, 801 N.E. 13th Street, Oklahoma City, OK 73104, USA; cassandra-querdibitty@ouhsc.edu (C.D.Q.); Marianna-Wetherill@ouhsc.edu (M.S.W.); Lancer-Stephens@ouhsc.edu (L.S.); 2 Department of Nutrition and Exercise Physiology, Elson S. Floyd College of Medicine, Washington State University Health Sciences Spokane, 412 E. Spokane Falls Blvd., Spokane, WA 99202, USA; bethany.williams1@wsu.edu; 3 Department of Nutritional Sciences, College of Allied Health, University of Oklahoma Health Sciences Center, 1200 N. Stonewall Ave., Oklahoma City, OK 73114, USA; Susan-Sisson@ouhsc.edu; 4 Department of Biostatistics and Epidemiology, Hudson College of Public Health, University of Oklahoma Health Sciences Center, 801 N.E. 13th Street, Oklahoma City, OK 73104, USA; Janis-Campbell@ouhsc.edu; 5 Department of Family & Preventive Medicine, College of Medicine, University of Oklahoma, 900 N.E. 10th Street, Oklahoma City, OK 73104, USA; Mary-Gowin@ouhsc.edu; 6 Institute for Research on Equity and Community Health (iREACH), ChristianaCare, Avenue North, 4000 Nexus Drive, CEI-300, Wilmington, DE 19803, USA

**Keywords:** Oklahoma, childcare, children’s environmental health, allergens, pesticides, chemicals, asthma, policy

## Abstract

Little is known about the environmental health-related policies and practices of early care and education (ECE) programs that contribute to childhood asthma, particularly in Oklahoma where child asthma rates (9.8%) and rates of uncontrolled asthma among children with asthma (60.0%) surpass national rates (8.1% and 50.3%, respectively). We conducted a cross-sectional survey with directors of Oklahoma-licensed ECE programs to assess policies and practices related to asthma control and to evaluate potential differences between Centers and Family Childcare Homes (FCCHs). Surveyed ECEs (*n* = 476) included Centers (56.7%), FCCHs (40.6%), and other program types (2.7%). Almost half (47.2%) of directors reported never receiving any asthma training. More Center directors were asthma-trained than FCCH directors (61.0% versus 42.0%, *p* < 0.0001). Most ECEs used asthma triggers, including bleach (88.5%) and air fresheners (73.6%). Centers were more likely to use bleach daily than were FCCHs (75.6% versus 66.8%, *p* = 0.04). FCCHs used air fresheners more than did Centers (79.0% versus 61.0%, *p* < 0.0001). The majority of ECEs (74.8%) used pesticides indoors. Centers applied indoor pesticides more frequently (i.e., monthly or more often) than did FCCHs (86.0% versus 58.0%, *p* < 0.0001). Policy, educational, and technical assistance interventions are needed to reduce asthma triggers and improve asthma control in Oklahoma ECEs.

## 1. Introduction

Early care and education (ECE) programs, also known as childcare programs, are critical environments that shape children’s health. Approximately 13 million (61%) children under the age of five in the United States (US) receive regular care in ECE programs [1], for as much as 50 h a week [2,3]. Young children attending ECE programs can be chronically exposed to environmental toxicants from commonly used products, such as cleaners, air fresheners, and pesticides, at ECEs [4,5,6,7,8,9]. These products may emit volatile organic compounds (VOCs) into the indoor air that can then interact with other chemicals to produce secondary pollutants known as particulate matter (PM) [10,11]. PM is a complex mixture of particles and liquid droplets including nitrates, sulfates, elemental and organic carbon, organic compounds (e.g., polycyclic aromatic hydrocarbons), biological compounds (e.g., endotoxin and cell fragments), and metals (e.g., iron, copper, nickel, zinc, and vanadium) in suspended air of various sizes of concern including “inhalable coarse particles” with a diameter of 2.5 to 10 µm (PM_10_), “fine particles” smaller than 2.5 µm in diameter (PM_2.5_), and “ultrafine particles” with a diameter less than 0.1 µm (PM_0.1_) [12,13]. Children’s small body size, unique behaviors and activity levels, physiology, and rapid development make them particularly vulnerable to these exposures and their potential health effects [14,15]. Children have higher respiratory rates, breathe a larger volume of air per unit body weight [16], and have increased physical exertion that results in greater inhalation of potentially harmful chemicals [17]. Childhood exposures to VOCs, PM, and nitrogen dioxide have been associated with adverse respiratory outcomes including decreased lung function, inflammation, and airway obstruction; increased allergen sensitization; and the exacerbation of asthma symptoms [15,18,19,20,21,22,23].

Environmental toxicants in indoor environments are dependent on numerous factors including the age [24] and structural quality of the building or facility [25,26,27,28], maintenance, and cleaning practices [10,29,30]. To illustrate, a building that is poorly maintained may be more susceptible to mold and pests potentially leading to the frequent use of pesticides [8], which were associated with higher indoor levels of VOCs [11,31,32,33]. While limited, studies have documented the presence of VOCs and PM (i.e., PM_2.5_ and PM_10_) in ECE settings [4,8,34]. A study in northern California [4] found several environmental toxicants in childcare facilities such as chloroform, benzene, and ethylbenzene, at estimated concentrations exceeding government health-based exposure levels. Those chemicals that reached exposure levels of concern were known carcinogens and endocrine disruptors. Additionally, these chemicals may exacerbate asthma and other respiratory illnesses and may disrupt neurocognitive functioning among children. Notably, a study of childcare centers in inner-city Washington D.C. [8], found a maximum concentration of commonly detected VOCs were over 14 times higher than those reported in the California study. Exposures to pest allergens such as cockroaches, mice, dust mites, and rodents have been associated with sensitization and are common triggers of childhood asthma symptoms [35,36,37,38]. A majority of ECEs in two North Carolina counties detected cockroach and mouse allergen, 52% and 83% respectively [39]. Similarly, a study in Arkansas, found 100% of Head Starts had detectable mouse allergens [40]. A combination of chemical and non-chemical pest control methods, also known as integrated pest management (IPM), aims to eliminate the source using pesticides and maintain low levels of pest allergens using less/non-toxic alternatives [41,42,43,44]. However, childcare centers revealed high management and staff turnover, hectic work environment, and inadequate coordination with cleaning and pest management contractors made it difficult to implement IPM strategies [45]. Organophosphorus and pyrethroids pesticides, known neurotoxins, are commonly detected indoors and [9] routinely applied in classrooms and on playgrounds [46]. Pesticide residues can remain on surfaces children frequently encounter (e.g., floors, carpets, furniture, toys) and accumulate in dust that can easily be inhaled or ingested by children [47].

ECE programs’ environmental health-related policies and practices may influence children’s exposures to environmental toxicants and subsequent risk of asthma attacks and adverse health outcomes. Understanding the types of policies and practices carried out by ECE programs is therefore paramount to creating healthier ECE environments. Yet, little is known about the environmental health-related policies and practices of ECE programs. Most studies to date with notable exceptions [8,48,49] were conducted in California [50,51,52,53], the state with the most protective policies, and most research was carried out in childcare centers (i.e., federally-supported Head Start programs and community-based childcare). Little research has been conducted in other geographical regions of the US or in family childcare homes (FCCHs) (i.e., home-based childcare). Centers and FCCHs’ environmental health-related policies and practices, including those related to childhood asthma control, may differ largely due to their setting and participation in accrediting bodies. Accrediting bodies, such as the National Association for the Education of Young Children (NAEYC), have standards that are generally more rigorous than those of state licensing requirements. The NAEYC has specific accreditation assessment items related to environmental health, including (1) air fresheners; (2) fragrance-free and least-toxic cleaning products; and (3) non-toxic pest management techniques (i.e., IPM) [54]. IPM is a safer approach to controlling pests because its strategies focus on preventing infestations by monitoring pests and limiting the use of harmful pesticides [55]. Regardless of the type of ECE program, all children should receive care in a healthy childcare environment that reduces asthma triggers and improves asthma control. 

The prevalence of childhood asthma in Oklahoma (9.8%) is higher than the national childhood asthma prevalence (8.1%) [56]. The rate of uncontrolled asthma among children with asthma in Oklahoma (60.0%) also exceeds the national rate (50.3%) [57]. In Oklahoma and in many other states, young children primarily receive childcare in one of two settings: Centers and FCCHs. Centers are usually located in larger facilities (i.e., commercial buildings), have multiple classes and larger sizes, and have multiple staff members [58]. FCCHs care for 12 or fewer children, are located in family homes or residences, and have a single provider, sometimes with an assistant [59].

The purpose of the present study was to characterize environmental health-related policies and practices of licensed ECE programs in Oklahoma caring for preschool-aged children (i.e., three to five years old). We examined policies and practices for Oklahoma ECE programs overall and also assessed differences between the two primary ECE program types: (1) Centers and (2) FCCHs. Our study focused on policies and practices in three key areas: (1) asthma training, prevention, and control; (2) cleaners and air fresheners; and (3) pesticides and pest control methods.

## 2. Materials and Methods

### 2.1. Study Design

The Communities and Classroom Health Survey, a cross-sectional survey of Oklahoma ECE directors, was conducted from November 2019 through February 2020. Eligible ECE programs were (1) licensed in Oklahoma and (2) cared for preschool-aged children. Our total sampling frame of existing Oklahoma-licensed ECE programs (*n* = 3121) included Head Starts (*n* = 343), community-based childcare (i.e., CBCC; *n* = 1130), and FCCHs (*n* = 1648). Program directors were targeted a priori as the primary survey participants; however, if preferred by the director, another staff member who was knowledgeable about the program’s policies or practices could participate. A total of 159 programs were excluded because (1) program approval was not obtained, (2) programs were Tribally-owned ECEs operated by a sovereign tribal nation with an Institutional Review Board, (3) programs did not care for preschool-aged children, or (4) programs were deemed to be duplicates. Of the 2962 programs that were eligible and invited to participate in the study, 191 were Head Starts, 1126 were CBCCs, and 1645 were FCCHs. Detailed methods on the survey and recruitment were previously published [60]. For the purpose of the current study, participating programs were categorized into one of two primary ECE types: (1) Center-based childcare or “Centers” (i.e., Head Starts and CBCCs) or (2) FCCHs. The OUHSC Institutional Review Board deemed this program-level study exempt. This was due to data being program-level, not individual-level; thus, this study was not considered as human-subjects research. 

### 2.2. Data Collection

Contact information was obtained from a current census of Oklahoma Head Start programs (provided by the Oklahoma Head Start Collaboration Office) and an up-to-date registry of CBCCs and FCCHs (provided by the Oklahoma Department of Health). In November 2019, eligible ECE programs were mailed a survey packet with (1) a cover letter with detailed consent instructions; (2) a survey booklet with instructions on how to complete the survey; (3) an optional link to complete the survey online using Research Electronic Data Capture (REDCap) [61,62]; and (4) a postage-paid business reply envelope for directors who preferred to complete the survey on paper. A reminder postcard was mailed to non-respondents in December 2019. A second and final round of survey packets was mailed out to non-respondents in January 2020. Reminder phone calls were made to non-respondents using publicly available telephone numbers in January and February 2020. Participants could enter a raffle for a $20 Amazon gift card for completing the survey. Identifiable information provided to participate in the raffle was deidentified and was not linked with survey responses. 

### 2.3. Measures

#### 2.3.1. Program Characteristics

Directors reported ECE program-level characteristics, including program type (i.e., Centers versus FCCHs). For analyses, ECE program type was collapsed into three categories: (1) Center (i.e., “center-based childcare” and “Head Start”), (2) FCCH (i.e., “family childcare home”), and (3) Other (i.e., “public pre-k program”). Public pre-k programs, which are typically a part of public-school systems and are privy to different policies and resources, were categorized as Other and were only included in analyses for the total sample. Programs were also classified by NAEYC accreditation status (yes/no). 

#### 2.3.2. Asthma-Related Training, Policies, and Practices

Items were adapted from the Preparing Asthma in Child Care (PACC) Instrument [63], which was designed to measure the preparedness of ECE programs to prevent and manage asthma exacerbations via asthma training for staff and asthma-related policies and guidelines. Types of asthma training assessed included (1) asthma basics (i.e., causes of asthma, signs of asthma flare-ups), (2) reducing asthma allergens and irritants, (3) asthma medication use and types, (4) asthma management plans, and (5) proper administration of asthma medications. Policies and guidelines assessed included (1) managing asthma medications and (2) reducing asthma allergens and irritants. Additional questions used to determine the presence of known asthma allergens included whether the program had: (1) any pets (e.g., cats, dogs, gerbils, or birds), (2) wall-to-wall carpet, and (3) staff that smoked or vaped on facility property. 

#### 2.3.3. Bleach and Air Fresheners

Items were also adapted from the Environmental Exposures in Child Care Facilities Study [4] to measure the usage of (1) bleach, (2) less toxic cleaners, and (3) air fresheners. 

#### 2.3.4. Pesticide Use and Pest Control

Items were adapted from the Pest Management and Pesticide Use in California Child Care Centers questionnaire to assess pest exposure, pest management, and pesticide use [64]. Indoor and outdoor pest problems in the past 12-months were measured using two questions with multi-select response options. Two questions were used to measure the frequency of pesticide use inside and outside the ECE facility in the past 12 months, including the use of pesticide sprays, scatters, and bombs. Two separate questions assessed who applied pesticides inside and outside of the ECE including multi-select response options. The presence of an ECE policy regarding pesticide use (when and how) was measured with a single question. Staff and parental notification prior to pesticide application inside or outside of the ECE were assessed by two questions. See Supplementary Material Figure S1 for a copy of all survey questions.

### 2.4. Data Analyses

Paper survey responses were entered into a REDCap database and combined with online survey responses. SAS version 9.4 (SAS Institute Inc., Cary, NC, USA) was used for all analyses. Descriptive statistics were conducted on all measures, including mean and standard deviations (*SD*s) for continuous variables and frequencies and percentages for categorical or nominal variables. Analyses were conducted with the total sample, then stratified by program type (i.e., Centers and FCCHs). The differences between Centers and FCCHs were evaluated with Chi-square analyses, Fisher’s exact tests for variables with more than 20% of cells with expected frequencies < 5, and *t*-tests for continuous variables. Statistical significance was set at *p* < 0.05. The initial significance level of 0.05 was adjusted to 0.025 for multiple hypothesis testing using the Bonferroni method [65]. 

Stratification by NAEYC accreditation, as a proxy to star rating, precluded analyses due to the small sample size (*n* = 58). A sensitivity analysis, however, was conducted to explore possible differences by NAEYC accreditation.

## 3. Results

### 3.1. Program Characteristics

**Figure 1:**
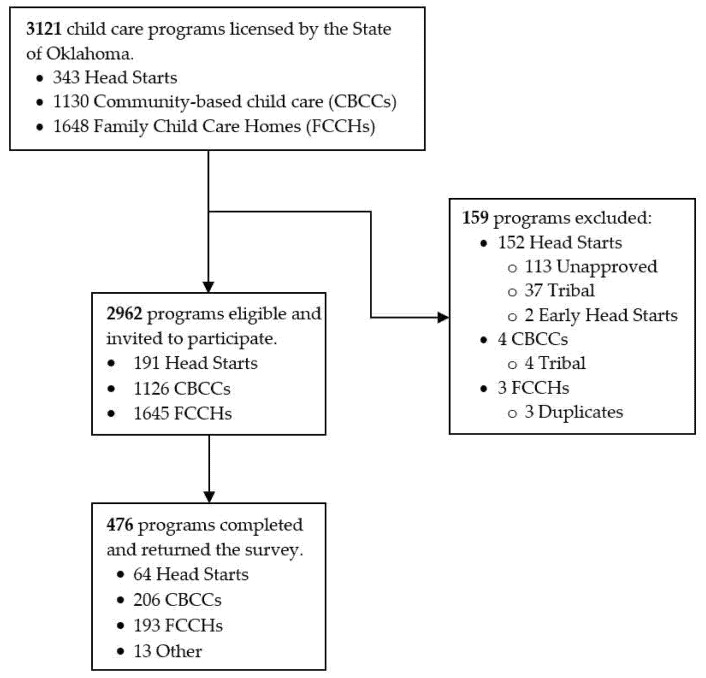
Program participation in the Communities and Classroom Health Survey (*n* = 476), Oklahoma 2019–2020.

The overall response rate was 16.0% and included 476 surveys (Figure 1), with 33.5% Head Starts, 18.3% CBCCs, and 11.7% FCCHs responding. 

Of the 476 participating ECE programs, a little over half (56.7%) were Centers (Table 1). Most programs (92.7%) were in operation for a full day and were fully enrolled (66.2%). Few programs were NAEYC accredited (12.2%). The average number of preschool classrooms was about two (*SD* = 2.4). Centers typically had about three preschool classrooms (*SD* = 2.8; Range:1–34) and FCCHs had about one (*SD* = 1.4; Range: 1–2). While Centers cared for an average of about 34 preschool-aged children (*SD* = 46.5; Range: 2–574), FCCHs had an average of fewer than four (*M* = 3.7; *SD* = 2.4; Range: 0–20).

### 3.2. Asthma-Related Training, Policies and Practices

About half (52.7%) of directors reported ever receiving any asthma training (Table 2). Center directors (61.0%) were more likely to report any asthma training than were FCCH directors (42.0%) (*p* < 0.0001), and were more likely to report training across all four training areas: (1) asthma basics (49.0% versus 36.0%, *p* = 0.01), (2) asthma medication use and types (37.0% versus 19.0%, *p* < 0.0001), (3) asthma management plans (17.0% versus 19.0%, *p* = 0.02), and (4) proper administration of asthma medications (42.0% versus 26.0%, *p* < 0.001). While most (82.4%) programs had policies/guidelines for managing asthma medications, Centers were more likely than FCCHs to have such policies/guidelines (88.0% versus 68.0%, *p* < 0.0001). Over one-in-four of programs (27.3%) overall had pets at their facilities. Over half of FCCHs (57%) had pets, while few (7%) Centers did (*p* < 0.0001). About one in ten (10.1%) programs reported that staff smoked or vaped on facility property. Centers, however, were more likely to report that staff smoked or vaped on facility property, albeit outside only, than were FCCHs (14.0% versus 5.0%, *p* < 0.001).

### 3.3. Bleach and Air Fresheners

Most (88.5%) programs reported using bleach at their facilities (Table 3), with Centers being more likely than FCCHs to use bleach daily or a few times a day (76.0% versus 67.0%, *p* = 0.04). A majority (62.0%) of programs reported that they used “low toxicity” or “less toxic” cleaners, with Centers less likely to use these cleaners than FCCHs (58.0% versus 68.0%, *p* = 0.03). A large proportion (73.6%) of programs reported using air fresheners. Centers were less likely to use air fresheners compared to FCCHs (61.0% versus 79.0%, *p* < 0.0001). Specifically, Centers were less likely to use scented candles (2.0% versus 19.0%, *p* < 0.0001), continuous-release air fresheners (19.0% versus 31.0%, *p* = 0.001), and essential oil diffusers (11.0% versus 20.0%, *p* = 0.01) than were FCCHs.

### 3.4. Pesticide Use and Pest Control

About a quarter (23.5%) of ECEs reported that pesticides were sprayed, scattered, or “bombed” inside their facility weekly or monthly (Table 4), with these applications more common in Centers than FCCHs (35.0% versus 6.0%, *p* < 0.001). About half (49.0%) of programs used a pest control company to apply pesticides inside their facilities. Centers used pest control companies for indoor pesticide applications more often than did FCCHs (64.0% versus 27.0%, *p* < 0.001). About one-in-six (15.6%) programs reported pesticide use outside their facility weekly or monthly. Centers reported more frequent outdoor pesticide applications than did FCCHs (20.0% versus 8.0%, *p* < 0.001). Less than half (43.3%) of programs used a pest control company for outdoor pesticide applications at their facilities, with Centers reporting doing so more often than FCCHs (51.0% versus 32.0%, *p* < 0.0001). A small proportion (21.8%) of ECE programs had a written policy about pesticide use. Centers were more likely than FCCHs to have a written pesticide use policy (31.0% versus 8.0%, *p* < 0.0001). Over one-in-three (36.7%) programs reported notifying parents before applying pesticides. Centers (29.0%), however, were less likely than FCCHs (42.0%) to notify parents before pesticide applications inside or outside their facility (*p* = 0.01). 

See Table 5 for more information about the ECE programs’ pest problems and pest control methods.

## 4. Discussion

Understanding the current environmental health policies and practices of ECEs is a critical first step to developing standardized policies and interventions to protect children’s health. Our study provides insight into the children’s environmental health-related policies and practices of Oklahoma ECEs and reveals some critical opportunities to improve: (1) asthma training, prevention, and control; (2) chemical cleaner and air freshener use; and (3) pest control methods and notification rules when chemical pesticides get used. Centers and FCCHs commonly reported several areas of concern for environmental exposure and asthma control, primarily exposure to potentially hazardous chemicals through routine use of bleach, air fresheners, and pesticides.

Our study revealed an important need for asthma-related training and program policies, and practices. Comparable with a study of 40 San Francisco, California centers [50], about half of directors reported receiving any asthma training, and even fewer reported having policies and guidelines for reducing asthma allergens and irritants. Center directors were more likely than FCCH directors to (1) receive asthma training and (2) report the presence of policies and guidelines for managing asthma medications. Asthma training for ECEs inclusive of asthma trigger avoidance education, particularly for FCCHs, may help ensure programs’ preparedness for the prevention and management of asthma flare-ups in this higher risk age group [23].

Our findings indicate that Oklahoma ECE programs routinely use cleaning products and air fresheners that release potentially harmful chemicals known to trigger asthma symptoms and cause adverse respiratory-related health outcomes [10,66,67,68,69,70,71]. Similar to findings from another study in Washington D.C. [8], most ECE programs in our study reported using bleach to clean their facilities. Centers were more likely to report daily or more frequent bleach use. Under Oklahoma Administrative Code 340:110-3-304. Cleanliness and Sanitation, ECE programs are required to use a household bleach solution or a sanitizer/disinfectant product with a US Environmental Protection Agency (EPA) registration number [58,59]. While effectively disinfecting and sanitizing is important for ECE health, products such as bleach pose risks to children’s respiratory health [66]. Alternative methods for sanitizing and disinfecting, such as fragrance-free, non-chlorine, hydrogen peroxide products that have less respiratory toxicity than do bleach or quaternary ammonias [72], may result in overall healthier environments for both children and providers. Additionally, there are national resources available to assist ECE programs in choosing safer disinfectants without compromising a hygienic environment including the (1) US EPA’s List N to identify EPA-registered products that can be used against emerging viral pathogens, such as the Coronavirus, not listed on the product label [73] and (2) the US Department of Labor-Occupational Safety and Health Administration’s Hazard Communication Standards to promote chemical safety at ECEs through the provision of information and training to staff regarding chemical labels, safety data sheets, and protocols outlining how to properly handle and store chemicals [74]. In contrast, we found higher rates of air freshener use than reported by Washington D.C. childcare providers [8] and childcare facilities that participated in the evaluation of the Children’s Environmental Health Network’s EcoHealthy Child Care Checklist [49]; use in FCCHs was particularly high. Air fresheners release more than 100 different hazardous chemicals, including VOCs (e.g., terpenes, such as limonene, alpha-pinene, and beta-pinene; terpenoids, such as linalool; and alpha-terpineol, such as formaldehyde, benzene, toluene, and xylene) and semi-volatile organic compounds (e.g., phthalates) that can contribute to indoor environmental exposure risk [10,67,68,69]. A pilot study of 14 childcare facilities in Washington D.C. detected six VOCs (i.e., benzene carbon, tetrachloride, chloroform, ethylbenzene, o-xylene, and toluene) inside most childcare facilities with detection frequencies ranging from 71% to 100% [8]. Currently, the Oklahoma Licensing Requirements for Child Care Programs [58,59] do not provide any guidance about air fresheners. Education about the potential hazards posed by ECE cleaning behaviors and safer cost-effective alternatives and standardized policies that limit the use of harmful cleaning products and air fresheners may reduce children’s exposures in ECE environments [48]. Technical guidance programs for ECEs that assist them in selecting the least toxic options for sanitizing and disinfecting and properly applying and storing products may help to reduce the potential impacts of such products on all children’s health.

Similarly, we found much higher usage of broad pesticide application methods (i.e., spray, scatter, or bomb) than reported from a survey conducted in California with 637 centers that found 47.0% of centers used sprays or foggers to mitigate pest problems, with 20.0% of those centers applying pesticides weekly or monthly [64]. Pesticides that are uncontained may become airborne and leave invisible residues on surfaces such as toys, shades, and walls that can remain for days, posing potential exposure and health risks to children. The majority of Centers in our study used pest control companies to apply pesticides inside their facilities and appeared to schedule the application of pesticides regularly. This practice is inconsistent with recommended IPM strategies to prevent and manage pest problems through removing entry points and access to water and food, only endorsing pesticide use as a last resort and then through contained methods. Pesticide applications inside ECE facilities where children spend most of their time increase their risk of exposure to hazardous residues that may cause exacerbations of asthma symptoms, pediatric cancers, and neurobehavioral and cognitive deficits [75]. Additionally, we found that Centers were less likely to notify parents before applying pesticides. Compared to FCCHs, Centers are housed in larger facilities in commercially zoned areas with more resources than FCCHs, which may explain the higher utilization of pest control companies. Our findings suggest pest control companies do not utilize IPM strategies in ECE environments and instead practice regularly scheduled applications of uncontained, chemical methods as part of their contracts with ECE programs. While California has the Healthy Schools Act [76], which seeks to reduce children’s exposures to potentially hazardous toxicants from pesticides in school and ECE environments, few other states including Oklahoma have policies or regulations for ECEs that adequately address children’s environmental health. To reduce young children’s exposures to pesticides in ECE environments, interventions should address IPM strategies and policies to better manage pests with an emphasis on the reduction of the overall cost of pest control methods with less/non-toxic alternatives. Importantly, adoption of effective pesticide use notification laws to protect the health and safety of employees and families of children attending the ECE.

Study strengths include participation from ECEs across the state and the involvement of both Centers and FCCHs, which enabled us to assess and compare policies and practices between these important, yet distinct, program types. The inclusion of FCCHs adds to the literature, as most research on ECE program environmental health-related policies and practices was conducted with Centers [50,51,77,78]. Few studies have examined these policies and practices among FCCHs [52]. To the best of our knowledge, none have assessed differences between Centers and FCCHs.

Study limitations to note include the use of self-reported data that precluded validation of the ECE program directors’ responses, which may be subject to social desirability and recall bias. Our study did not objectively measure indoor environmental exposures to the reported chemicals used by ECE programs such as potential exposures to ultrafine and fine particles directly emitted or formed secondarily due to indoor air chemistry. Thus, we cannot determine whether the exposures exceed US EPA and the World Health Organization’s indoor air quality guidelines [79]. Although our sample size was moderate, the overall response rate was low, especially for FCCHs, which make up the bulk of the state’s licensed ECE programs. It is possible that ECE programs that chose not to participate in this study may be systematically different from those that did and therefore may not represent the overall ECE or FCCH population in Oklahoma. Finally, since we did not include some tribally-owned and operated ECE programs, our study does not adequately assess policies and programs in ECEs owned and operated by sovereign tribal nations in the state. Future researchers may wish to include (1) observations of ECEs and program practices; (2) in-depth data collection about ECE programs’ written policies, practices, products, and chemicals used; and (3) measurements of air quality and chemical exposures.

Outside of California [76], few states have policy requirements for ECEs designed to reduce children’s exposure to chemicals and other environmental toxicants or to address exposures to environmental toxicants that may originate from or near the ECE facility [80]. Some professional accrediting organizations, such as the NAEYC, have more standards aimed at protecting children’s environmental health. NAEYC requires accredited ECE programs to (1) test for radon, (2) use IPM strategies for pest control, and (3) maintain an allergen-free facility [54]. Since only a small (12.2%) portion of participating ECE programs in our study reported NAEYC accreditation, we were unable to assess whether NAEYC-accredited programs’ environmental policies and practices differed from those of programs that were not accredited due to the small sample size.

## 5. Conclusions

This study is the first of its kind in Oklahoma and is among very few conducted outside of California and Washington D.C. that have assessed ECE programs’ environmental health-related policies and practices. It is the first to evaluate differences in these policies and practices between Centers and FCCHs. The findings highlight the need for interventions that will improve rates of asthma training, reduce the presence of asthma triggers, and promote the use of less/non-toxic pest control methods such as IPM. Given the many differences we found in policies and practices between Centers and FCCHs, it is critical that interventions be tailored to these contexts and address the most salient drivers of organizational and/or provider behaviors in these settings. Interdisciplinary collaboration among clinicians, public health professionals, education and child development specialists, and ECE providers themselves may enhance future research to further understand the drivers and impacts of environmental exposures in ECE programs and develop interventions to promote healthier ECE environments in both Centers and FCCHs.

## Figures and Tables

**Table 1:** Early care and education program characteristics (*n* = 476), Oklahoma 2019–2020.

	Total ^1^ (*n* = 476)	Center ^2^ (*n* = 270)	FCCH ^3^ (*n* = 193)	
Program Characteristics	*n* (%)	*n* (%)	*n* (%)	*p*-value ^4^
Program Hours				
Half day	24 (5.0%)	19 (7.1%)	3 (1.6%)	-
Full day	441 (92.7%)	248 (92.9%)	187 (98.4%)	**0.01**
Fully Enrolled				
Yes	315 (66.2%)	166 (61.5%)	141 (73.1%)	-
No	161 (33.8%)	104 (38.5%)	52 (26.9%)	**0.01**
NAEYC Accreditation				
Yes	58 (12.2%)	43 (15.9%)	15 (7.8%)	-
No	418 (87.8%)	227 (84.1%)	178 (92.2%)	**0.01**
Program Characteristics	Mean (*SD*)	Mean (*SD*)	Mean (*SD*)	*p*-value ^5^
Number of Classrooms				
Total classrooms	4.2 (3.8)	5.9 (3.9)	1.6 (1.7)	**<0.001**
Preschool aged classrooms	2.1 (2.4)	2.6 (2.8)	1.3 (1.4)	**<0.001**
Number of Children				
Total children	43.7 (52.3)	67.9 (57.0)	8.8 (4.1)	**<0.001**
Preschool-aged children	21.9 (38.5)	34.4 (46.5)	3.7 (2.4)	**<0.001**

^1^ Total includes community-based early care and education programs, Head Starts, public pre-k, and other programs unspecified or undetermined; ^2^ Center includes community-based early care and education programs and Head Starts; ^3^ FCCH = family childcare home. ^4^ Chi-square tests to analyze differences between Centers and FCCHs; Bolded numbers are *p-*values < 0.05. ^5^ *t*-tests to analyze differences between Centers and FCCHs; Bolded numbers are *p-*values < 0.05.

**Table 2:** Asthma-related training, policies, and practices reported for all early care and education programs, Centers, and family childcare homes (FCCHs), Oklahoma 2019–2020.

Survey Items	Total (*n* = 476)	Center (*n* = 270)	FCCH (*n* = 193)	
	*n* (%)	*n* (%)	*n* (%)	*p*-Value ^1^
Ever received any type of asthma training				
Yes	251 (52.7%)	166 (61.0%)	81 (42.0%)	-
No	225 (47.3%)	104 (39.0%)	112 (58.0%)	**<0.0001**
Type of asthma training received				
Asthma basics	-	-	-	-
Yes	204 (42.9%)	131 (49.0%)	70 (36.0%)	-
No	272 (57.1%)	139 (51.0%)	123 (64.0%)	**0.01**
Reducing asthma allergens and irritants	-	-	-	-
Yes	88 (18.5%)	58 (21.0%)	28 (15.0%)	-
No	388 (81.5%)	212 (79.0%)	165 (85.0%)	0.06
Asthma medications use and types	-	-	-	-
Yes	139 (29.2%)	99 (37.0%)	37 (19.0%)	-
No	337 (70.8%)	171 (63.0%)	156 (81.0%)	**<0.0001**
Asthma management plans	-	-	-	-
Yes	65 (13.7%)	46 (17.0%)	18 (9.0%)	-
No	411 (86.3%)	224 (83.0%)	175 (91.0%)	**0.02**
Proper administration of asthma medications	-	-	-	-
Yes	170 (35.75%)	116 (43.0%)	50 (26.0%)	-
No	306 (64.3%)	154 (57.0%)	143 (74.0%)	**<0.0001**
Policies/guidelines about managing asthma medications				
Yes	378 (82.4%)	237 (88.0%)	132 (68.0%)	-
No	81 (17.7%)	33 (12.0%)	61 (32.0%)	**<0.0001**
Policies/guidelines about reducing asthma allergens and irritants				
Yes	175 (36.8%)	109 (40.0%)	63 (33.0%)	-
No	301 (63.2%)	161 (60.0%)	130 (67.0%)	0.09
Pets present at facility				
Yes	130 (27.3%)	19 (7.0%)	110 (57.0%)	-
No	346 (72.7%)	251 (93.0%)	83 (43.0%)	**<0.0001**
Facility has wall-to-wall carpet				
Yes	120 (25.2%)	60 (22.0%)	58 (30.0%)	-
No	356 (74.8%)	210 (78.0%)	135 (70.0%)	0.06
Staff smoke or vape on facility property				
Yes, but outside only	48 (10.1%)	39 (14.0%)	9.0 (5.0%)	**<0.0001**
Yes, both inside and outside	0 (0.0%)	0 (0.0%)	0 (0.0%)	-
No	428 (89.9%)	231 (86.0%)	184 (95.0%)	-

^1^ Chi-square tests to analyze differences between Centers and FCCHs; Bolded numbers are *p-*values < 0.05.

**Table 3:** Reported use of bleach, air fresheners, and cleaners for all early care and education programs, centers, and family childcare homes (FCCHs), Oklahoma 2019–2020.

Survey Items	Total (*n* = 476)	Center (*n* = 270)	FCCH (*n* = 193)	
	*n* (%)	*n* (%)	*n* (%)	*p*-Value ^1^
Use bleach at facility				
Yes	421 (88.5%)	233 (86.0%)	178 (92.0%)	-
No	55 (11.6%)	37 (14.0%)	15 (8.0%)	0.05
Frequency of bleach use at facility				
Every few months or less often	-	-	-	-
Yes	15 (3.2%)	6 (2.2%)	7 (3.6%)	-
No	461 (96.9%)	264 (97.8%)	186 (96.4%)	0.37
Monthly or a few times a month	-	-	-	-
Yes	17 (3.6%)	8 (3.0%)	9 (4.7%)	-
No	459 (96.4%)	262 (97.0%)	184 (95.3%)	0.34
Weekly or a few times a week	-	-	-	-
Yes	49 (10.3%)	15 (5.6%)	33 (17.1%)	-
No	427 (89.7%)	255 (94.4%)	160 (82.9%)	**<0.0001**
Daily or a few times a day	-	-	-	-
Yes	340 (71.4%)	204 (75.6%)	129 (66.8%)	-
No	136 (28.6%)	66 (24.4%)	64 (33.2%)	**0.04**
Use “low toxicity” or “less toxic” cleaners at facility				
Yes	295 (62.0%)	156 (58.0%)	131 (68.0%)	-
No	181 (38.0%)	114 (42.0%)	62 (32.0%)	**0.03**
Use air fresheners at facility				
Yes	323 (73.6%)	164 (61.0%)	153 (79.0%)	-
No	116 (26.4%)	106 (39.0%)	40 (21.0%)	**<0.0001**
Types of air fresheners used				
Scented candles	42 (8.8%)	6 (2.0%)	36 (19.0%)	**<0.0001**
Spray air fresheners	190 (39.9%)	106 (39.0%)	81 (42.0%)	0.56
Continuous release (such as a plug-in)	112 (23.5%)	52 (19.0%)	59 (31.0%)	**<0.0001**
Incense	6 (1.3%)	2 (1.0%)	4 (2.0%)	0.24
Essential oils (reed diffuser or other type of diffuser)	73 (15.3%)	34 (13.0%)	36 (19.0%)	0.07
Essential oils electric or battery diffuser	71 (14.9%)	31 (11.0%)	39 (20.0%)	**0.01**
Potpourri	1 (0.2%)	0 (0.0%)	1 (1.0%)	0.42
Gel canister	10 (2.1%)	6 (2.0%)	3 (2.0%)	0.74
Scented wax melts	25 (5.3%)	10 (4.0%)	15 (8.0%)	0.06
Other types	13 (2.7%)	5 (2.0%)	8 (4.0%)	0.14

^1^ Chi-square tests to analyze differences between Centers and FCCHs; Bolded numbers *p*-values < 0.05; Fisher’s exact tests to analyze differences between Centers and FCCHs for variable with a cell size < 5; Initial significance level of 0.05 was adjusted to 0.025 for multiple hypothesis testing using the Bonferroni method.

**Table 4:** Reported pest problems, pest control methods, applicators, frequency, and related topics for all early care and education programs, centers, and family childcare homes (FCCHs), Oklahoma 2019–2020.

Survey Items	Total (*n* = 476)	Center (*n* = 270)	FCCH (*n* = 193)	
	*n* (%)	*n* (%)	*n* (%)	*p*-Value ^1^
Have indoor pest problem(s)				
Yes	225 (47.3%)	142 (53.0%)	78 (40.0%)	-
No	251 (52.7%)	128 (47.0%)	115 (60.0%)	**0.01**
Have outdoor pest problem(s)				
Yes	271 (56.9%)	153 (57.0%)	114 (59.0%)	-
No	205 (43.1%)	117 (43.0%)	79 (41.0%)	0.61
Use pesticides indoors				
Do not use pesticides				
Yes	120 (25.2%)	37 (14.0%)	82 (42.0%)	-
No	356 (74.8%)	233 (86.0%)	111 (58.0%)	**<0.0001**
Pesticides applied by pest control company	-	-	-	-
Yes	233 (49.0%)	173 (64.0%)	52 (27.0%)	-
No	243 (51.1%)	97 (36.0%)	141 (73.0%)	**<0.0001**
Pesticides applied by director or someone else	-	-	-	-
Yes	104 (21.9%)	45 (17.0%)	57 (30.0%)	-
No	372 (78.2%)	225 (83.0%)	136 (70.0%)	**<0.0001**
Use pesticides outdoors				
Do not use pesticides	-	-	-	-
Yes	121 (25.4%)	53 (20.0%)	66 (34.0%)	-
No	355 (74.6%)	217 (80.0%)	127 (66.0%)	**<0.0001**
Pesticides applied by pest control company	-	-	-	-
Yes	206 (43.3%)	137 (51.0%)	62 (32.0%)	-
No	270 (56.7%)	133 (49.0%)	131 (68.0%)	**<0.0001**
Pesticides applied by director or someone else	-	-	-	-
Yes	149 (31.3%)	79 (29.0%)	68 (35.0%)	-
No	327 (68.7%)	191 (71.0%)	125 (65.0%)	0.17
Frequency of pesticide used indoors				
Pesticides were used, but not sprayed, scattered, or “bombed”	-	-	-	-
Yes	168 (35.3%)	61 (23.0%)	104 (54.0%)	
No	308 (64.7%)	209 (77.0%)	89 (46.0%)	**<0.0001**
Weekly or monthly	-	-	-	-
Yes	112 (23.5%)	94 (35.0%)	12 (6.0%)	-
No	364 (76.5%)	176 (65.0%)	181 (94.0%)	**<0.0001**
Yearly or a few times a year	-	-	-	-
Yes	117 (24.6%)	67 (25.0%)	50 (26.0%)	-
No	359 (75.4%)	203 (75.0%)	143 (74.0%)	0.79
Whenever pests become problem	-	-	-	-
Yes	49 (10.3%)	26 (10.0%)	22 (11.0%)	-
No	427 (89.7%)	244 (90.0%)	171 (89.0%)	0.54
Frequency of pesticide used outdoors				
Pesticides were used, but not sprayed, scattered, or “bombed”	-	-	-	-
Yes	153 (32.1%)	74 (27.0%)	77 (40.0%)	-
No	323 (67.9%)	196 (73.0%)	116 (60%)	**<0.0001**
Weekly or monthly	-	-	-	-
Yes	74 (15.6%)	54 (20.0%)	16 (8.0%)	-
No	402 (84.5%)	216 (80.0%)	177 (92.0%)	**<0.0001**
Yearly or a few times a year	-	-	-	-
Yes	143 (30.0%)	74 (27.0%)	67 (35.0%)	-
No	333 (70.0%)	196 (73.0%)	126 (65.0%)	0.09
Whenever pests become a problem	-	-	-	-
Yes	81 (17.0%)	53 (20.0%)	27 (14.0%)	-
No	395 (83.0%)	217 (80.0%)	166 (86.0%)	0.11
Written policy for pesticide use ^2^				
Yes	87 (21.8%)	73 (31.0%)	12 (8.0%)	-
No	313 (78.3%)	164 (69.0%)	138 (92.0%)	**<0.0001**
Staff are notified before pesticides are applied ^3^				
Yes	274 (71.7%)	166 (72.0%)	103 (75.0%)	-
No	108 (28.3%)	66 (28.0%)	35 (25.0%)	0.52
Parents are notified before pesticides are applied ^4^				
Yes	129 (36.7%)	69 (29.0%)	59 (42.0%)	-
No	223 (63.4%)	166 (71.0%)	80 (58.0%)	**0.01**

^1^ Chi-square tests to analyze differences between Centers and FCCHs; Bolded numbers are *p*-values < 0.05; ^2^ *n* = 76 Not applicable, no pesticides are used (missing; omitted from analysis); *n* = 94 Not applicable, no pesticides used (missing; omitted from analysis); ^3^ *n* = 94 Not applicable, no pesticides used (missing; omitted from analysis); ^4^ *n* = 90 Not applicable, no pesticides are used (missing; omitted from analysis).

**Table 5:** Reported pest problems, pest control methods, applicators, frequency, and related topics (*n* = 476) Oklahoma 2019–2020.

Survey Items	Indoors		Outdoors	
	*n*	(%)	*n*	(%)
Type of pest problems (listed in alphabetical order)				
None	203	(42.7%)	159	(33.4%)
Ants	125	(26.3%)	131	(27.7%)
Aphids	0	(0.0%)	2	(0.4%)
Bed bugs	7	(1.5%)	0	(0.0%)
Cockroaches	61	(12.8%)	6	(1.3%)
Crickets	5	(1.1%)	0	(0.0%)
Fleas	9	(1.9%)	14	(2.9%)
Head lice	55	(11.6%)	0	(0.0%)
Mold	6	(1.3%)	0	(0.0%)
Mosquitoes	0	(0.0%)	17	(3.6%)
Rodents	38	(8.0%)	18	(3.8%)
Scorpions	0	(0.0%)	6	(1.3%)
Snails/Slugs	3	(0.6%)	6	(1.3%)
Snakes	0	(0.0%)	6	(1.3%)
Spiders	88	(18.5%)	99	(20.8%)
Termites	4	(0.8%)	5	(1.1%)
Ticks	0	(0.0%)	3	(0.6%)
Wasps/Yellow Jackets	5	(1.1%)	147	(30.9%)
Other Pests	15	(3.2%)	19	(30.9%)
Pest control methods used				
Nothing used	104	(21.9%)	109	(22.9%)
Other methods	10	(2.1%)	8	(1.7%)
Chemical Pest Control Methods Used	-	-	-	-
Sprayed Pesticides	220	(46.2%)	224	(47.1%)
Poison pellets or powders	7	(1.5%)	17	(3.6%)
Moth balls	4	(0.8%)	0	(0.0%)
Applied weed killer	0	(0.0%)	60	(12.6%)
Non-chemical Pest Control Methods Used	-	-	-	-
Cleaned the area	151	(31.7%)	79	(16.6%)
Sealed cracks/openings	76	(16.0%)	35	(7.4%)
Removed food sources	62	(13.0%)	16	(3.4%)
Mouse or rat traps	53	(11.1%)	13	(2.7%)
Bait Stations or poison traps	53	(11.1%)	27	(5.7%)
Fixed leaks	26	(5.5%)	11	(2.3%)
Sticky fly strips	22	(4.6%)	12	(2.5%)
Installed screens or other barriers	14	(2.9%)	11	(2.3%)
Cut grass or weeds	0	(0.0%)	147	(30.9%)
Who applies pesticides at facility				
No one, nothing used	120	(25.2%)	121	(25.4%)
Pest control company	233	(49.0%)	206	(43.3%)
Me (participant her/himself)	54	(11.3%)	62	(13.0%)
Property owner	18	(3.8%)	30	(6.3%)
Custodial/janitorial staff	18	(3.8%)	37	(7.8%)
Facility director	16	(3.4%)	12	(2.5%)
Another staff member	7	(1.5%)	12	(2.5%)
Someone else (Other)	4	(0.8%)	5	(1.1%)
Not Sure	3	(0.6%)	4	(0.8%)
A family member	2	(0.4%)	5	(1.1%)
Frequency of pesticide use				
Nothing used	152	(31.9%)	141	(29.6%)
Once a week	2	(0.4%)	4	(0.8%)
Once a month	110	(23.1%)	71	(14.9%)
Once a year	26	(5.5%)	43	(9.0%)
A few times a year	91	(19.1%)	101	(21.2%)
Whenever pests become a problem	49	(10.3%)	81	(17.0%)
Pesticides were used, but not sprayed, scattered, or “bombed”	17	(3.6%)	13	(2.7%)

## Data Availability

The data presented in this study are available on request from the corresponding author. The data are not publicly available.

## Supplementary Materials

The following are available online at https://www.mdpi.com/article/10.3390/ijerph18168491/s1, Figure S1: Communities and Classroom Health Survey.
