# Supplementary material for: Environmental Health-Related Policies and Practices of Oklahoma Licensed Early Care and Education Programs: Implications for Childhood Asthma
Source: Int J Environ Res Public Health. 2021 Aug 11;18(16):8491. doi: 10.3390/ijerph18168491 (PMC8391589; doi:10.3390/ijerph18168491)
Supplement: Supplementary file 1 [file ijerph-18-08491-s001.zip › ijerph-1279592-supplementary.pdf]

Figure S1. Communities and Classroom Health Survey.

1. Which of the following best describes your center?

	YES	NO
Center based child care	◯	◯
Family childcare home	◯	◯
Head Start	◯	◯
Public pre-k program	◯	◯
Other (*please specify*): ____________________	◯	◯

2. Which of the following best describes your center (*select all that apply*)?

Half-day
Full-day
Other (*please specify*):

____

3. Is your center NAEYC accredited [1]?

Yes
No
Not sure

4. Is your center fully enrolled at this time?

Yes
No
Not sure

5. What is the number of classrooms in your center **total**?

____

6. What is the number of classrooms in your center **with children who are 3-5 years old**?

____

7. What is the number of children in your center **total**?

____

8. What is the number of children in your center **who are 3-5 years old**?

____

9. Does your center have policies and guidelines for managing asthma medications?
- ◯ Yes
- ◯ No
- ◯ Not sure

10. In the past 12 months, did you keep any pets such as cats, dogs, gerbils, or birds at your center?
- ◯ Yes
- ◯ No

11. Does your center have wall-to-wall carpet?
- ◯ Yes
- ◯ No

12. Does anyone who works at your center smoke or vape on your center's property?
- ◯ Yes, but OUTSIDE only
- ◯ Yes, both INSIDE and OUTSIDE
- ◯ No

[1] NAEYC: National Association for the Education of Young Children.

13. Does your center have policies and guidelines for reducing asthma allergens and irritants?

- ◯ Yes
- ◯ No
- ◯ Not sure

14. Have you ever received any training on any of the following aspects of asthma (*select all that apply*)?

- ☐ No, I've never received any type of asthma training
- ☐ Asthma basics (causes of asthma, signs of asthma flare-ups)
- ☐ Reducing asthma allergens and irritants
- ☐ Asthma medication use and types
- ☐ Asthma management plans
- ☐ Proper administration of asthma medications

15. How often do you use bleach at your center?

- ◯ Never, we do not use bleach
- ◯ Daily or a few times a day
- ◯ Weekly or a few times a week
- ◯ Monthly or a few times a month
- ◯ Every few months or less often

16. Does your center use low toxicity or less toxic cleaners?

- ◯ Yes
- ◯ No
- ◯ Not sure

17. What kind of air fresheners are used at your center (*select all that apply*)?

- ☐ None – do not use any type of air freshener or candle
- ☐ Scented candles
- ☐ Spray air fresheners
- ☐ Continuous release (like a plug-in)
- ☐ Incense
- ☐ Essential oils (reed diffuser or other type of diffuser)
- ☐ Essential oil electric or battery diffuser
- ☐ Potpourri
- ☐ Gel canister
- ☐ Other types of air freshener(s) (*please specify*): ____________________

18. Does your center have a written policy for use of pesticides (bug killers, weed killers, rat killers, etc.), stating when and how to apply them?

- ◯ Yes
- ◯ No
- ◯ Not sure
- ◯ Not applicable, **no** pesticides are used

19. Are staff notified before pesticides (including weed killers) are going to be applied **<u>INSIDE or OUTSIDE</u>** of your center?

- ◯ Yes
- ◯ No
- ◯ Not applicable, **no** pesticides are used

20. Are parents notified before pesticides (including weed killers) are going to be applied **<u>INSIDE or OUTSIDE</u>** of your center?

- ◯ Yes
- ◯ No
- ◯ Not applicable, **no** pesticides are used

21. In the past 12 months, which of the following pests were a problem **INSIDE** your center (i.e., INDOORS) *(select all that apply)*?

☐ None- **did not** have any **INDOOR** pest problems.

☐ Ants ☐ Fleas ☐ Termites ☐ Snails/slugs ☐ Mold

☐ Cockroaches ☐ Rodents ☐ Aphids ☐ Spiders ☐ Head lice

☐ Bed bugs ☐ Other pest(s) *(please specify):* ______________________

22. In the past 12 months, which of the following pest control methods did your center use for controlling pests **INSIDE** your center (i.e., INDOORS) *(select all that apply)*?

Nothing used ☐ Sprayed Pesticides ☐ Bait stations or poison traps

☐ Sticky fly strips ☐ Mouse or rat traps ☐ Moth balls

☐ Poison pellets or powders ☐ Removed food sources ☐ Fixed leaks

☐ Cleaned the area ☐ Sealed cracks/openings ☐ Installed screens or other barriers

☐ Other *(please specify):* ______________________

23. In the past 12 months, who applied the **INDOOR** pesticides used at your center *(select all that apply)*?

Nothing used

☐ Myself ☐ Director ☐ Another staff member

☐ Property owner ☐ Custodial/janitorial staff ☐ Pest control company

☐ Not sure ☐ Other *(please specify):* ______________________

24. In the past 12 months, how frequently were pesticides sprayed, scattered, or "bombed" **INSIDE** your center (i.e., INDOORS) *(select all that apply)*?

Nothing used ☐ Once a week ☐ Once a month

☐ Once a year ☐ A few times a year ☐ Whenever pests become a problem

☐ Not applicable- pesticides were used, **but not** sprayed, scattered, or "bombed"

25. In the past 12 months, which of the following pests were a problem **OUTSIDE** your center (i.e., OUTDOORS) *(select all that apply)*?

- ☐ None- **did not** have any **OUTDOOR** pest problems.
- ☐ Ants
- ☐ Fleas
- ☐ Termites
- ☐ Snails/slugs
- ☐ Mold
- ☐ Cockroaches
- ☐ Rodents
- ☐ Aphids
- ☐ Spiders
- ☐ Scorpions
- ☐ Wasps/yellow jackets
- ☐ Other pest(s) *(please specify):* ______________________

26. In the past 12 months, which of the following pest control methods did your center use for controlling pests **OUTSIDE** your center (i.e., OUTDOORS) *(select all that apply)*?

- Nothing used
- ☐ Sprayed Pesticides
- ☐ Bait stations or poison traps
- ☐ Sticky fly strips
- ☐ Mouse or rat traps
- ☐ Applied weed killer
- ☐ Poison pellets or powders
- ☐ Removed food sources
- ☐ Fixed leaks
- ☐ Cleaned the area
- ☐ Sealed cracks/openings
- ☐ Installed screens or other barriers
- ☐ Cut grass or weeds
- ☐ Other pest(s) *(please specify):* ______________________

27. In the past 12 months, who applied the **OUTDOOR** pesticides used at your center *(select all that apply)*?

- Nothing used
- ☐ Myself
- ☐ Director
- ☐ Another staff member
- ☐ Property owner
- ☐ Custodial/janitorial staff
- ☐ Pest control company
- ☐ Not sure
- ☐ Other *(please specify):* ______________________

28. In the past 12 months, how frequently were pesticides sprayed, scattered, or "bombed" **OUTSIDE** your center (i.e., OUTDOORS) *(select all that apply)*?

- Nothing used
- ☐ Once a week
- ☐ Once a month
- ☐ Once a year
- ☐ A few times a year
- ☐ Whenever pests become a problem
- ☐ Not applicable- pesticides were used, **but not** sprayed, scattered, or "bombed"
